# The alignment between phenotypic plasticity, the major axis of genetic variation and the response to selection

**DOI:** 10.1098/rspb.2015.1651

**Published:** 2015-10-07

**Authors:** Martin I. Lind, Kylie Yarlett, Julia Reger, Mauricio J. Carter, Andrew P. Beckerman

**Affiliations:** 1 Department of Animal and Plant Sciences, University of Sheffield, Western Bank, Sheffield S10 2TN, UK; 2 Animal Ecology, Department of Ecology and Genetics, Uppsala University, Uppsala 752 36, Sweden; 3 Centro Nacional del Medio Ambiente, Universidad de Chile, Avenida Larrain 9975, La Reina, Santiago, Chile

**Keywords:** *Daphnia pulex*, *Chaoborus flavicans*, *Gasterosteus aculeatus*, genetic accommodation, phenotypic plasticity, predation

## Abstract

Phenotypic plasticity is the ability of a genotype to produce more than one phenotype in order to match the environment. Recent theory proposes that the major axis of genetic variation in a phenotypically plastic population can align with the direction of selection. Therefore, theory predicts that plasticity directly aids adaptation by increasing genetic variation in the direction favoured by selection and reflected in plasticity. We evaluated this theory in the freshwater crustacean *Daphnia pulex*, facing predation risk from two contrasting size-selective predators. We estimated plasticity in several life-history traits, the G matrix of these traits, the selection gradients on reproduction and survival, and the predicted responses to selection. Using these data, we tested whether the genetic lines of least resistance and the predicted response to selection aligned with plasticity. We found predator environment-specific G matrices, but shared genetic architecture across environments resulted in more constraint in the G matrix than in the plasticity of the traits, sometimes preventing alignment of the two. However, as the importance of survival selection increased, the difference between environments in their predicted response to selection increased and resulted in closer alignment between the plasticity and the predicted selection response. Therefore, plasticity may indeed aid adaptation to new environments.

## 1. Introduction

Phenotypic plasticity is the ability of a genotype to produce more than one phenotype depending upon the environment [1]. The value of adaptive phenotypic plasticity is that it generates environment-specific phenotypes that are similar to what would be expected by a locally adapted specialist to that environment [1,2], thus reflecting what would be favoured by selection. Therefore, adaptive plasticity, it is argued, can influence local adaptation by producing phenotypes that are pre-adapted to the new environment. The role of plasticity in local adaptation has experienced a recent upsurge in interest due to several important models that predict how plasticity may enable survival in novel and extreme environments long enough for genetic change to take place through a process called genetic accommodation [3–6].

Empirical plasticity research, however, remains largely focused on detecting genetic variation in plasticity of single or pairs of traits varying across environments. This narrow focus is even true for recent theory where models of adaptation linked to phenotypic plasticity typically focus on single plastic traits [4,6,7]. However, it has long been recognized that the genetic variance and covariance of multiple traits can be expressed in an environment-specific manner [8–11], and that selection acts on the variance and covariance among traits [12–14]. A plastic response thus often involves a set of traits responding in concert [8,15–17] and requires a multivariate view of plasticity [16,18,19]. Such a multivariate perspective of plasticity can be captured in a **G** matrix, the matrix of additive genetic variance and covariance among traits [8]. It is well known that exposure to extreme environments can release cryptic genetic variation and affect the genetic architecture of traits [20,21], but plasticity may also be restricted to phenotypic changes with no effect on the underlying genetic architecture at all, in which case plasticity may sometimes buffer against evolutionary change [7].

Draghi & Whitlock [22] presented a model that deals with both the long-standing expectation that plasticity aids adaptation and the multivariate nature of plasticity. Linking developmental genetics, plasticity and adaptation, their theory predicts that plasticity can directly aid adaptation by increasing genetic variation along the lines of least resistance, known as *g*_max_ (*sensu* Schluter [23]), the major axis of genetic variation estimated from the **G** matrix. Using a simulation approach based upon a developmental gene network model, they recovered the well-established understanding that adaptive phenotypic plasticity evolves in a heterogeneous environment with reliable cues [2]. Importantly, however, their model resulted in an increase in genetic variance and covariance (and also mutational variance) in the direction of greatest divergence between the environments, which with stable selection gradients would be the direction most favoured by selection. Draghi & Whitlock [22] suggest that the plastic response to multivariate selection predisposes the developmental machinery to, and increases the genetic variance in, the direction of most divergence between the environments, as long as plasticity is adaptive and selection is sufficiently strong along *g*_max_. In this context, it is predicted that plasticity would align the phenotype to the major axis of genetic variation and, ultimately, be in line with the direction of selection. However, the theory has an underlying theoretic assumption of a stable **G** matrix, an assumption that can be challenged [24–26].

We present a multivariate, experimental evaluation of this theory, and the assumption of a stable **G** matrix [22], using a model system of phenotypic plasticity: predator-induced defences in the water flea, *Daphnia pulex*. Specifically, we evaluate whether phenotypic plasticity can align a phenotype with the major axis of genetic variation (*g*_max_) and whether, ultimately, plasticity aligns a phenotype with the predicted response to selection [22]. We do so with *D. pulex*, which responds to predator chemical cues with inducible plastic and adaptive changes in morphology, life history and behaviour [27–30]. We focus on life-history plasticity, a major form of predator-induced plasticity in daphnids [29–32] and one benefiting from substantial evolutionary theory linked to size-selective predation [33].

## 2. Methodology

### (a) Species and study populations

Our work centres on 19 genotypes (clones) of *D. pulex* (Cladocera), a microcrustacean with a cosmopolitan distribution, and a keystone herbivore of algae in ponds and lakes [34]. In the UK, it is subjected to contrasting and seasonal predation pressure by young-of-the-year fish in spring, and midge larvae during summer and autumn [35]. Fish are visual predators and prefer large *Daphnia*, which gives a benefit to individuals that mature early and at a small size [32,36]. By contrast, midge larvae are gape-limited, and therefore prey upon small juvenile *Daphnia*, favouring growth to reach the size refuge and mature at a larger size [29,32,37].

*Daphnia pulex* were collected in May and June 2009 in northern England from the ponds LD3 and LD6 in Cumbria, and Crabtree in Yorkshire, from which 5, 7 and 7 clones (*n* = 19), respectively, were identified using 17 microsatellite markers (ponds, clones and markers are described in the electronic supplementary material, table S1)*.* The clones were maintained in the laboratory in hard artificial pond water (ASTM [38]) under a 16 L : 8 D cycle and fed the algae *Chlorella vulgaris*.

### (b) Kairomone extraction to generate plasticity

To generate predator-induced responses, we isolated kairomones from both fish and midge larvae. Water enriched in fish kairomones was generated following Beckerman *et al.* [32,39] by housing two similar-sized (4–6 cm) three-spined sticklebacks *Gasterosteus aculeatus* (fed commercially available frozen *Daphnia*) in 6 l artificial pond water at 12.5°C in a controlled temperature room for at least 24 h, whereafter the water was filtered through a 47 mm filter (Fisherbrand, Fisher Scientific) and a 0.45 µm pore-sized filter (Sartorius Stedim Biotech). Midge kairomone was isolated via filtration and solid-phase extraction from frozen *Chaoborus flavicans* (Honka, Germany) following the protocol of Tollrian [31] (see also [32,35,39]).

### (c) Life-table experiment and trait plasticity

We generated plasticity in four life-history traits, estimated from a standard life-table experiment: age and size at maturity, fecundity and somatic growth rate of adults. We defined age at maturity as the age when eggs were first released into the brood pouch, size at maturity as the linear distance between the top of the head and the base of the tail at age at maturity, and adult growth rate as the log-change in body size between the first and third clutch, divided by the time between these clutches. Fecundity was estimated as the number of eggs produced in the first three clutches and used to calculate the intrinsic population growth rate (*r*). Using data of three clutches is considered appropriate in *D. pulex* where Riessen & Sprules ([40], fig. 4) showed that the first three clutches can explain approximately 94% of total *r*.

These data were collected from daphnids housed in a temperature-controlled laboratory set to 21°C and a 16 L : 8 D cycle. To avoid maternal and grand-maternal effects, the clones were grown for three generations under experimental food conditions prior to the experiment. We initiated the experiment by exposing third-generation mothers between their second and third brood to the predation-risk treatment conditions, and the experimental animals used in the life-table experiments were neonates from their third brood. Each mother was placed in a 60 ml glass jar filled with experimental medium containing 49 ml ASTM [38], food (*C. vulgaris*, 2 × 10^5^ cells ml^−1^) and marinure (a liquid seaweed extract for micronutrients, Wilfrid Smith Ltd., Northans, UK, 0.018 µml^−1^), and were selected for experiment when holding black-eyed embryos (12 h prior to parturition).

The predator cue treatments were constructed either by adding 0.5 µl ml^−1^ of the concentrated midge kairomone to experimental vessels or by replacing 20% of the ASTM with ASTM enriched with fish kairomones. These cue concentrations give strong induction of morphological and life-history traits and correspond to realistic predation pressures [17,32,39,41]. The neonates were exposed to the predator cue treatment during their whole development with water conditions reset daily by moving the individuals into fresh treatment medium (ASTM, food, marinure and kairomones).

Life-history traits were estimated under experimental conditions and individuals were photographed daily to collect size-specific data on each replicate. The experiment started when one neonate from each mother was placed in a cylindrical glass tube (height: 150 mm, diameter: 19 mm) filled with 30 ml treatment medium. When reaching maturity, the adults were housed in 60 ml glass jars filled with 50 ml treatment medium. The experiment was terminated when the adults reached their third clutch. Each of the 19 clone × treatment combinations was replicated 2–5 times (mean 3.8), giving a total sample size of 143 individuals (71 in the fish and 72 in the midge cue treatment).

Reproductive fitness was calculated as the intrinsic population growth rate (*r*) in the absence of predation mortality (e.g. risk cues only). We calculated *r* for each replicate by solving the Euler–Lotka equation on life-table data spanning first instar to third clutch [42].

### (d) Phenotypic plasticity, selection gradients and the response to selection

Here we present how we (i) quantify plasticity and define ‘vectors of plasticity’ associated with predation risk, (ii) quantify the genetic covariance matrix and it's major axis of variation, and then (iii) construct a composite selection gradient of survival and reproduction. We then detail how we (iv) generate predicted, predator-specific multivariate responses to selection from (ii) and (iii). This represents the raw material with which to evaluate Draghi & Whitlock's [22] hypotheses and is summarized in electronic supplementary material, figure S1.

#### (i) Phenotypic plasticity and the multivariate vector of plasticity

We evaluated plasticity, genetic variation in plasticity and estimated the vector of plasticity using a Bayesian MCMC mixed model. We fitted a model with predator treatment as fixed effect and a random-regression specification for the random effect where each clone possessed its own intercept and slope. We estimated trait plasticity via the fixed effect. Genetic variation in plasticity is estimated by evaluating whether the slopes terms in the random effects specification is significant. All tests were evaluated via 95% credible intervals from the joint posterior distribution of fixed and random effects. We also compared, using DIC (the Bayesian equivalent to AIC), a model with and without the random slopes term, as an additional test of genetic variation in plasticity.

We then defined the vector of trait plasticity to midge and to fish predation risk for each of size at maturity, age at maturity and adult growth rate. The vector of plasticity is the ‘distance moved by each trait’ and was estimated from the treatment-specific mode of the trait values. Specifically, from the joint posterior distribution of trait values in each treatment, we constructed the joint posterior distribution of the mode of each vector.

We also here estimated the angle separating the vector of plasticity to each predator, *θ*_plast_, defined by the cosine formula for a dot product [43]:2.1
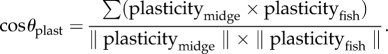


Fecundity (*r*) was not included in the estimation of plasticity and was reserved for the analysis of selection gradients (below).

The models for each trait were fitted in R [44] using *MCMCglmm* [45] with source pond (*n* = 3) as a fixed effect and clone (*n* = 19) as a nested random effect, allowing for different intercepts and slopes. We used parameter-expanded priors, and models were fitted with a burn-in of 50 000 and sampling that produced 1000 estimates of the joint posterior distribution from more than 500 000 iterations of the chains. All models were checked for autocorrelation in the chains.

#### (ii) Estimating **G** matrices and *g*_max_

We next estimated the predator treatment-specific variance–covariance matrices (**G**) for the three traits. We used a Bayesian MCMC multivariate mixed model (*MCMCglmm* [45]) to estimate **G** following [19],2.2
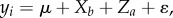
where *y_i_* is a vector of trait values in treatment *i* and *Z_a_* is a design matrix relating individuals to total clonal genetic effects *a*, which estimates an unstructured **G** matrix of the genetic effects. All trait data from the life-table experiment was first centred and scaled to s.d. = 1.

As above, source pond (*n* = 3) was a fixed effect and clone the random effect. The clone random effect was specified as an unconstrained variance–covariance matrix of the total genetic (clonal) variance and covariance among the three traits.

*g*_max_ is the first principal component of **G** and the major axis of genetic variation. Using the tools presented in [19] for estimating *g*_max_ and angles separating *g*_max_ from Bayesian MCMC mixed models, we derived a joint posterior distribution of *g*_max_, and the angle separating *g*_max_ between the two predation treatments via [43],2.3
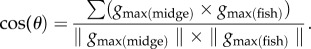


#### (iii) Estimating the selection gradient *β*

Selection under predation risk in nature depends both upon reproduction and survival. Their relative importance depends upon the predation regime in ponds, which fluctuates over the season [35]. Therefore, we specified the following five selection gradients, each representing a different weighting of reproduction (*β*_R_) and survival (*β*_S_): *β*_R_, *β*_R_ + 0.5*β*_S_, *β*_R_ + *β*_S_, 0.5*β*_R_ + *β*_S_ and *β*_S_.

The linear selection gradient *β*_R_ was estimated as the regression coefficients from multiple linear regression of traits against intrinsic population growth rate (*r*) (see life-table experiment above). As we were interested in comparing the alignment between the response to selection and the plasticity, fitness was centred and scaled to unit variance, to give comparable strength of selection between the predation treatments, only allowing the direction of selection to vary. These analyses were implemented using parametric methods from the *rsm* package [46] in R [44]. As all traits were measured on the same individuals, we used randomization to test the significance of all parameters of *β*_R_, taking the potential non-independence of residuals into account [47].

The selection gradients on survival, *β*_S_ were defined from the empirical and theoretical literature of size-selective predation on *Daphnia.* This literature (see [33] for theory) is strongly focused around assumptions that gape-limited predators, such as *Chaoborous* larvae, target small prey [37,48,49], while visually hunting predators such as fish instead target large prey [36,50]. To reflect this, we defined the *β*_S_ coefficient for size at maturity to −1 in the fish treatment (highest fitness for small *Daphnia*) and to +1 in the midge treatment (highest fitness for large *Daphnia).* All other coefficients were set to zero in this instance.

Finally, to create the composite selection gradients, *β*_R_ and *β*_S_ were standardized to a total length (strength of selection) of 1. We then created the composite selection gradients, representing a several combinations of reproduction (*β*_R_) and survival (*β*_S_): *β*_R_, *β*_R_ + 0.5*β*_S_, *β*_R_ + *β*_S_, 0.5*β*_R_ + *β*_S_ and *β*_S_. The resulting composite selection gradients were also standardized to a length of 1, to enable meaningful comparisons of the response to selection in the following step.

#### (iv) Predicting the multivariate response to selection

We next estimated the vector of multivariate response to selection of size at maturity, age at maturity and adult somatic growth rate using the multivariate breeders equation, Δ*z* = **G***β* [51].

Using the **G**-matrices estimated for each treatment and the five composite selection gradients, we estimated Δ*z* (2**G** × 5*β* = 10 Δ*z*). Because **G** is a joint posterior distribution of 1000 estimates (see above), multiplying **G** by *β* generates a joint posterior distribution of Δ*z* for each composite selection gradient and treatment. We thus propagated the variability in our estimates of **G** (captured in the posterior distribution of **G**) to Δ*z* [19,52]. We then estimated the angles between the treatment-specific responses to selection for each *β*, as defined in equation (2.3) above.

### (e) Alignment between plasticity, *g*_max_ and response to selection

The above methods provide the raw material (see the electronic supplementary material, figure S1) to test the hypothesis that plasticity aids adaptation. From above, we have posterior distributions describing (i) the vectors of plasticity (how far a trait moves) and the angle between the plastic response, (ii) the vectors of *g*_max_ (major axis of genetic variation) and the angle between these major axes, and (iii) the predicted response to the five combinations of survival and reproductive selection, Δ*z*, in each treatment and the angle between these responses.

Our analyses rely on testing whether the angle between vectors (e.g. between a vector of plasticity and Δ*z*) are significantly different. Ovaskainen *et al*. [52] and Robinson & Beckerman [19] make clear that while calculating the angle is straightforward, a test of whether it is significant requires a special test statistic. Following Robinson & Beckerman [19] and Ovaskainen *et al.* [52], multiple samples of the posterior provide a way to generate multiple estimates of *θ*, which can be used to compare the difference in angle within each group to differences in angle between each group [19,52]:
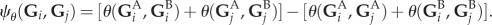


Each and every one of our angle comparisons can be calculated by applying this formula to the posterior distributions of each metric, resulting in a statistical test of whether angles deviate or align. We formally define alignment as a non-significant angle difference within treatments. This definition is characterized as a ‘match’ between types of responses.

However, this definition of alignment is sensitive to low power, as alignment is acceptance of the null hypothesis. Therefore, we also evaluated alignment via mismatch [53] where we predict that vectors that are aligned within treatments are significantly mis-aligned between treatments. We calculated the mismatch between the predator-specific *g*_max_/Δ*z* and the plastic trait expression induced by the wrong predator (e.g. the angle between *g*_max(fish)_ and plasticity_midge_). A non-significant angle of alignment combined with a significantly different angle of mismatch is the strongest inference about alignment, as it tests our power to reject the null hypothesis.

## 3. Results

### (a) Plasticity

**Figure 1.**
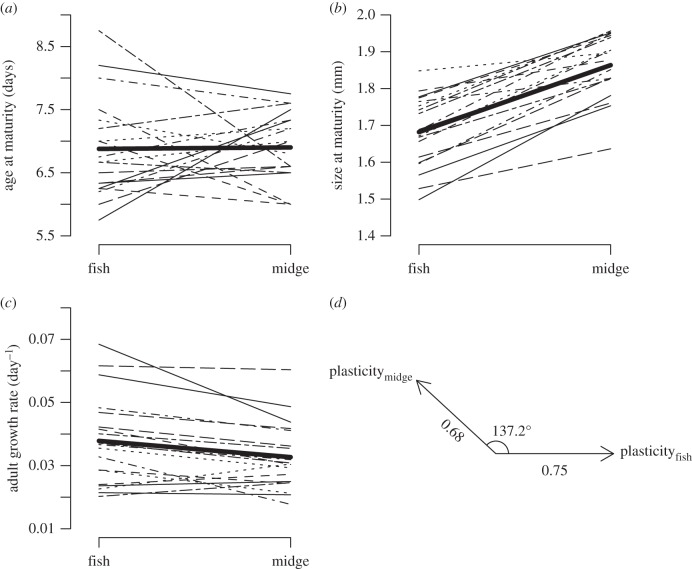
Reaction norms, based upon clone means, for predator cue-specific expression of (*a*) age at maturity, (*b*) size at maturity and (*c*) adult growth rate. (*d*) The angle between the vectors of multi-trait plasticity. Bold lines represent the mean clone response; for posterior modes see results section.

*Daphnia pulex* showed plastic changes between predator treatments (figure 1; electronic supplementary material, table S2). Exposure to midge kairomones resulted in larger size at maturation (midge: 1.87 mm; fish: 1.68 mm; pMCMC < 0.001) and a lower adult growth rate (midge: 0.034 day^−1^; fish: 0.040 day^−1^; pMCMC = 0.008) than did exposure to fish kairomones. Mean age at maturation did not differ between the treatments (midge: 7.12 days; fish: 7.07 days; pMCMC = 0.751). We did not include a control treatment, but both fish and midge cues are known to result in plastic trait induction relative to a control [32]. The vector of multi-trait plasticity was constructed using the full posterior distribution of the scaled and centred trait inductions, and the posterior modes for the traits in the midge treatment vector were 0.048 (age), 0.649 (size) and −0.159 (adult growth), while the corresponding trait modes for the fish treatment vector were −0.062 (age), −0.623 (size) and 0.167 (adult growth). These plasticity vectors differed between the treatments by an angle of 137.2° (95% CI: 100.4°–172.5°, *p* < 0.001). Surprisingly, among these genotypes, we found no significant genetic variation in plasticity in size at maturation (−0.026, 95% CI: −0.17–0.08) and adult growth rate (−0.11, 95% CI: −0.37–0.08), the two plastic traits, confirmed by very similar DIC values between models with and without the random slope term (size at maturation: DIC_intercept_ = 279, DIC_intercept+slope_ = 280; adult growth rate: DIC_intercept_ = 296, DIC_intercept+slope_ = 296). By contrast, we found significant genetic variation for plasticity in age at maturation, the not significantly plastic trait (−0.49, 95% CI: −1.04 to −0.07, DIC_intercept_ = 389, DIC_intercept+slope_ = 365). In summary, in classical **G** × **E** terms, we have **E** effects on two traits but no **G** × **E** (parallel reaction norms with non-zero slopes), and **G** × **E** in one trait, but no **E** (crossing reaction norms, but zero slope on average).

### (b) The **G** matrix and *g*_max_

We found significant broad sense heritability for all traits (age at maturity, size at maturity, adult growth rate) in both predator cue treatments (electronic supplementary material, table S3). Moreover, we found a significant and strong negative genetic covariance between size at maturity and adult growth rate in the fish treatment. The genetic correlation between size at maturity and adult growth rate was negative in both treatments, but stronger in the fish cue treatment, where we also found a positive genetic correlation between age and size at maturity.

*g*_max_ explained the same amount of genetic variation in each treatment (fish: 60.08%; midge: 56.04%; electronic supplementary material, table S4). The angle between *g*_max_ of the two treatments was 66.6° (95% CI: 42.3°–86.2°), indicating a substantial plastic rotation of the **G** matrix between the two predator treatments (electronic supplementary material, figure S2). This rotation was true in 94% of the posterior distribution samples used to test the angle differences, suggesting *p* = 0.06 [52]. We found no difference in the total amount of clonal genetic variation (fish: 1.16, CI: 0.89–1.80; midge: 1.10, CI: 0.72–1.61) between the treatments.

### (c) Selection gradients

In both predator cue treatments, we found that early age at maturity, large size at maturity and high adult growth rate all resulted in high reproductive fitness (electronic supplementary material, tables S5 and S6), resulting in very similar *β*_R_ between the treatments, as opposed to the divergent selection gradients for size-selective survival *β*_S_ (electronic supplementary material, figure S3). From *β*_R_ and *β*_S_, we then created the five composite selection gradients used for predicting the response to selection (electronic supplementary material, table S7).

### (d) Predicted response to selection

**Figure 2.**
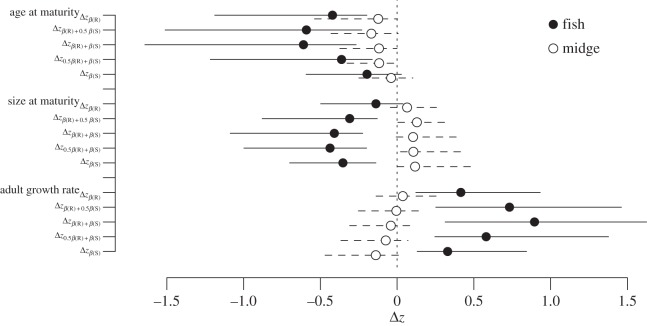
The predicted response to selection (Δ*z*) using composite selection on reproduction (Δ*z_*β*_*_(R)_), survival (Δ*z_*β*_*_(S)_) or reproduction and survival combined to selection in the fish (black circles, solid lines) and midge (white circles, dashed lines) cue treatment. The horizontal lines correspond to the 95% HPD interval.

Figure 2 and electronic supplementary material, figure S4, tables S8 and S9 present the range of predicted responses to selection across the combinations of *β*_R_ and *β*_S._ The angles between responses to each predator range from 48.9° to 137.83°, increasing as the importance of selection on survival is weighted more and more heavily. Selection on survival was required to detect a significant difference in the response to selection by each predator, but even weighting this by 50% of its strength results in approximately 30° rotation between responses. Despite the differences in selection gradients for reproduction and survival in the fish treatment (positive selection on size for reproduction, negative for survival, resulting in a difference in angle of 118.8°), the predicted response to selection was remarkably similar for all combinations of *β*_R_ and *β*_S_ (figure 2; electronic supplementary material, table S9).

### (e) Alignments

#### (i) Fish-induced plasticity, *g*_max_ and Δ*z*

We found strong alignment between the vector of plasticity and *g*_max_. There was no significant difference in the angle between the vector of multi-trait plasticity and *g*_max_ (table 1); plasticity aligned with the predicted response to selection, Δ*z*, when selection was based entirely on survival. It did not align with plasticity in any combination of selection gradients that involved *β*_R_ (table 1).

**Table 1.** Tests for alignment between plasticity (the vector of plastic trait inductions), the direction of maximum genetic variation (*g*_max_) and the response to selection on reproduction (Δ*z_*β*_*_(R)_), survival (Δ*z_*β*_*_(S)_) or reproduction and survival combined. Angle is expressed as the posterior mode with 95% CI. Significant angle differences are indicated by asterisks. Non-significant angles indicate alignment.

treatment	comparison	angle	*p*	alignment
fish	plasticity, *g*_max_	51.39° (19.93–143.41°)	0.248	yes
	plasticity, Δ*z_*β*_*_(R)_	60.43° (27.46–98.28°)	0.003*	—
	plasticity, Δ*z*_*β*(R)+0.5*β*(S)_	55.38° (22.20–89.76°)	0.004*	—
	plasticity, Δ*z_*β*_*_(R)+*β*(S)_	53.77° (19.47–86.43°)	0.014*	—
	plasticity, Δ*z*_0.5*β*(R)+*β*(S)_	44.62° (14.15–83.00°)	0.014*	—
	plasticity, Δ*z_*β*_*_(S)_	43.80° (10.75–78.81°)	0.085	yes
midge	plasticity, *g*_max_	97.85° (60.71–143.82°)	0.025*	—
	plasticity, Δ*z_*β*_*_(R)_	65.73° (21.31–103.55°)	0.044*	—
	plasticity, Δ*z_*β*_*_(R)+0.5*β*(S)_	52.81° (19.32–96.59°)	0.073	yes
	plasticity, Δ*z_*β*_*_(R)+*β*(S)_	49.29° (19.21–95.47°)	0.068	yes
	plasticity, Δ*z*_0.5*β*(R)+*β*(S)_	56.09° (18.26–90.86°)	0.066	yes
	plasticity, Δ*z_*β*_*_(S)_	63.09° (13.57–95.15°)	0.081	yes

#### (ii) Midge-induced plasticity, *g*_max_ and Δ*z*

Midge-induced plasticity and Δ*z* and plasticity and *g*_max_ were aligned in all instances where the composite selection gradient involved some degree of survival selection (table 1).

#### (iii) Validation via testing for misalignment

We validated our power to detect misalignment (a significant angle difference) by comparing the treatment-specific *g*_max_ and Δ*z* with the wrong plasticity vector. For both predator cue treatments, we detected a significant misalignment for all combinations of *β*, indicating that we have the power to detect misalignment. However, for *g*_max,_ we found alignment with the wrong plasticity vector (no significant angle difference) in both comparisons (electronic supplementary material, table S10).

## 4. Discussion

The role of phenotypic plasticity in adaptation and diversification continues to be a central focus in evolutionary ecology. Theory on how plasticity evolves, how it might aid in adaptation to novel, rapidly changing environments and how it might influence diversification is replete. Data, however, are often lacking to evaluate the emerging ideas. Draghi & Whitlock [22] proposed a new addition to this arsenal of theory, suggesting that phenotypic plasticity evolves to align phenotypic responses with the major axis of genetic variation. This idea, captured in an elegant model of development, provides a theoretical framework for the simple idea that plasticity can ‘pre-adapt’ populations to selection regimes, as long as the novel environment is an extension of the environment inducing the plastic response. Here, we present a multivariate, experimental evaluation of this idea using a classic system to study phenotypic plasticity: predator-induced defences in the water flea, *D. pulex*.

### (a) Alignment with plasticity of the traits

In a simulation study, Draghi & Whitlock [22] showed that selection on phenotypic plasticity could result in genetic correlations among the traits, such that genetic variance is increased in the direction of the plastic trait expression which, with stable selection gradients, also is the direction favoured by selection.

For perfect alignment between plasticity and the response to selection, the theory assumes a stable **G** matrix across environments where *g*_max_ and plasticity should be aligned. This would aid adaptation in the direction of plastic trait expression. Moreover, for plasticity to align with the response to selection, the different contributions to the selection gradient (i.e. reproduction and survival) should be in a similar direction. Otherwise, alignment depends upon the relative importance of reproduction and survival selection.

Predator cues in our experiments resulted in major differences in the **G** matrix between treatments, suggesting that genetic variance can be expressed in an environment-specific fashion [11,16]. However, environmental-specific changes in the **G** matrix go outside the assumptions of [22], whose model assumes **G** to be stable across environments, which complicates predictions.

We found a very pronounced difference in plastic trait expression (137.2°) between the fish and the midge cue treatment, while the difference in *g*_max_ between the treatments was substantial but smaller (66.6°). This difference in angles separating plasticity and *g*_max_ suggests a higher degree of shared genetic architecture between environments, possibly constraining adaptation in the directions of plastic trait induction. Therefore, we would not predict alignment between *g*_max_ and plasticity in both predator cue treatments, and indeed only *g*_max_ in the fish cue treatment aligned with the plastic trait induction. Furthermore, as *g*_max_ differed much less than plasticity, *g*_max_ in the midge cue treatment was actually more aligned with the plastic trait induction in the fish cue treatment, rather than in its own treatment. Together this suggests that shared genetic architecture between environments can limit alignment between plasticity and *g*_max_.

Given the interest in the role of plasticity for adaptation [3,6], our data show that the **G** matrix is environment-specific, potentially aiding adaptation, but that the shared genetic architecture between environments can result in greater plasticity in traits than in their underlying genetic variance and covariance. This potentially constrains adaptation in the direction of a plastic response, as divergence often [24] (but not always [26]) follows *g*_max_, at least in the short term. Nevertheless, the fact that the **G** matrix is environmental-specific is beneficial for adaptation to different environments.

However, adaptation is the response to selection, which also depends upon the environment-specific selection gradient. While similar trait values were important for high reproduction in both environments, survival in the midge cue treatment selects for large size [37,48,49], while small size is beneficial in the fish cue treatment [36,50]. Thus, especially for the fish treatment, the trait combinations selected for by survival or reproduction differ markedly.

Therefore, we predicted that the degree of alignment between the plastic trait induction and the predicted response to selection would depend upon the relative contribution of survival and reproduction to the composite selection gradient. Indeed, the degree of alignment was dependent upon the nature of selection, and increased in both environments with increased contribution of survival selection. This was most marked in the fish cue treatment, where these selection gradients were most dissimilar.

The lack of alignment with predicted selection on reproduction (but not survival) could indicate that the induced life-history plasticity in *D. pulex* is mainly a survival benefit, a suggestion that has strong empirical support [28–30]. This suggests that if predators are the main selective agent affecting *D. pulex*, then the response to selection will be aligned with anti-predator plasticity, despite a **G**-matrix constrained by shared genetic architecture between the two environments. Therefore, phenotypic plasticity, as predicted by Draghi & Whitlock [22], can move populations towards phenotypic adaptive peaks that would be generated by selection.

### (b) Power to detect alignment

Recalling that alignment is acceptance of the null hypothesis, we followed the approach of [53] to also test alignment with the wrong plasticity vector. We found that the angle between Δ*z* and the predator-induced plasticity was always smaller than the angle between Δ*z* and the plasticity vector induced by the wrong predator. Moreover, we were always able to detect significant misalignment between Δ*z* and the wrong plasticity vector, suggesting that we have power to detect misalignment if present. This is important given the low number of surviving individuals of some clones and the resulting variance in their genetic estimates.

Nevertheless, we suggest that alignment should be seen as continuous rather than a categorical observation based on a single significance value. Except for plasticity and *g*_max_ in the midge cue treatment, all angles are less than 90°, suggesting that they are clearly not in the wrong direction. It may be more important to consider the degree of (mis-) alignment, and the Bayesian MCMC approach to estimating angle changes provides a robust route to this end [19,52]. Additionally, our assessment of (mis-)alignment is also contingent on how survival and reproduction selection gradients combine. Appropriate, empirically derived weighting of these two portions of selection is necessary for more precise system-specific conclusions about how (mis-)alignment might shape the response to selection.

### (c) Environmental-specific changes in the **G** matrix

Novel or stressful environments can release cryptic genetic variation or change the genetic architecture of traits [20,21]. However, the suggestion that environmental cues may induce plastic change in the genetic variance and covariance of traits [54] has only lately attracted attention from experimental biologists [19]. Our data reveal one of very few empirical examples of such induced **G** matrix plasticity (see also [9–11]).

We show predator chemical cues can induce changes in the **G** matrix of life-history traits. This is a necessary response to invoke adaptive arguments associated with plasticity, but has rarely been shown. Environmental-specific change in the **G** matrix was, however, not modelled by [22], which makes any direct test of their model predictions complicated in the current study. The **G** matrix is, however, known to evolve over evolutionary time [24,25], for example following divergence [12–14,26], which together with several findings of environment-specific changes in **G** [9–11] suggests that it is sensitive to environmental input. We suggest that this sensitivity is not random but can follow the plasticity of the traits.

### (d) Predicted response to selection

To predict the response to selection, we used five combinations of the two fundamental selection gradients, one based upon reproduction in the presence of predator kairomones and one based upon the extensive literature on size-selective survival/mortality in the face of size-selective predation [36,37,50].

These composite gradients and several features of our data influenced our calculation of response to selection to each predator. The selection gradients for reproductive fitness were identical, which was in stark contrast to the different **G** matrices in the two predator cue treatments, where the covariance differences drive the response to selection. The selection gradient analysis revealed that in both treatments, highest reproductive fitness would be obtained by maturing early and at a large size, and to continue to grow after maturity. As there is a strong correlation between size and number of eggs that fit in the brood pouch [28], it is hardly surprising that these trait combinations result in high reproductive fitness. Therefore, it may be surprising that the predicted response to selection differed between the treatments despite identical selection gradients on reproduction. However, differences in indirect selection caused by plastic changes in genetic covariance structure are responsible for the divergent responses, as there was strong opposing indirect selection in the fish treatment for small size at maturation, and indirect selection in the midge treatment for low adult growth rate.

This should be contrasted to the strikingly different selection gradients on survival in the face of real predators. In midge predation, large size is beneficial because *Chaoborus* midge larvae are gape-limited predators [37]. By contrast, when visually hunting fish are present, highest survival is obtained by having a small size [36,50]. Thus, for the fish treatment, the selection gradients for survival and reproduction were markedly different, because large size at maturation, which is adaptive in terms of reproduction, is maladaptive in terms of survival in the presence of large size-selective, visually hunting fish.

Despite these differences, the predicted response to selection was qualitatively similar for all composite selection gradients within each treatment, but strikingly different for each predator cue treatment, with increased differences with increased contribution of survival selection to the overall selection gradient. The predicted response to selection in the fish treatment was to mature early, at a smaller size, and to have high adult growth rate, while the predicted response in the midge treatment was to mature at a larger size. The conclusion here is that the indirect selection, linked to predator-specific covariance among traits, matters.

## 5. Conclusion

Draghi & Whitlock [22] suggest that phenotypic plasticity may evolve to align phenotypic responses with the major axis of genetic variation, which could result ultimately in alignment of plasticity and the response to selection. We tested this elegant idea in a multivariate, experimental evaluation of using a classic system to study phenotypic plasticity: predator-induced defences in the water flea, *D. pulex.* Our results suggest that a multivariate picture of plasticity, articulating variance and covariance changes among environments, and the response to selection to multiple pressures, are vital to understanding the generality of this long-standing idea. Our focus on *Daphnia*, which can be locally adapted to the types of predators present in a lake, either by selection by the predators themselves [27,30,55,56] or indirectly through seasonal predation and temperature changes [57], indicates that plastic changes in the genetic variance and covariance of traits may play an unexplored role in diversification of populations, especially if selection is mainly driven by survival, but that the response could be somewhat constrained by shared genetic architecture. Plastic changes in genetic architecture goes outside the predictions of the model [22], but could aid adaptation in the direction of the plastic response.

## Supplementary Material

Supplementary material

## Data accessibility

Data are available from Dryad (http://dx.doi.org/10.5061/dryad.8t89m); core functions for the Bayesian MCMC based analyses are available from GitHub at https://github.com/andbeck/mcmc-plus-tensor.

## References

[1] DeWitt TJ, Scheiner SM 2004 Phenotypic plasticity: functional and conceptual approaches. New York, NY: Oxford University Press.

[2] Tollrian R, Harvell CD (eds). 1998 The ecology and evolution of inducible defenses. Princeton, NJ: Princeton University Press.

[3] West-Eberhard MJ 2003 Developmental plasticity and evolution. New York, NY: Oxford University Press.

[4] Lande R 2009 Adaptation to an extraordinary environment by evolution of phenotypic plasticity and genetic assimilation. J. Evol. Biol. 22, 1435–1446. (10.1111/j.1420-9101.2009.01754.x) 19467134

[5] Lande R 2015 Evolution of phenotypic plasticity in colonizing species. Mol. Ecol. 24, 2038–2045. (10.1111/mec.13037) 25558898

[6] Chevin L-M, Lande R, Mace GM 2010 Adaptation, plasticity, and extinction in a changing environment: towards a predictive theory. PLoS Biol. 8, e1000357 (10.1371/journal.pbio.1000357) 20463950 PMC2864732

[7] Price TD, Qvarnström A, Irwin DE 2003 The role of phenotypic plasticity in driving genetic evolution. Proc. R. Soc. Lond. B 270, 1433–1440. (10.1098/rspb.2003.2372) PMC1691402 12965006

[8] Via S, Lande R 1987 Evolution of genetic variability in a spatially heterogeneous environment: effects of genotype-environment interaction. Genet. Res. Camb. 49, 147–156. (10.1017/S001667230002694X) 3596235

[9] Sgrò CM, Hoffmann AA 2004 Genetic correlations, tradeoffs and environmental variation. Heredity 93, 241–248. (10.1038/sj.hdy.6800532) 15280897

[10] Brock MT, Weinig C 2007 Plasticity and environment-specific covariates: an investigation of floral-vegetative and within flower correlations. Evolution 61, 2913–2924. (10.1111/j.1558-5646.2007.00240.x) 17941839

[11] Robinson MR, Wilson AJ, Pilkington JG, Clutton-Brock TH, Pemberton JM, Kruuk LEB 2009 The impact of environmental heterogeneity on genetic architecture in a wild population of Soay sheep. Genetics 181, 1639–1648. (10.1534/genetics.108.086801) 19204380 PMC2666526

[12] Cano JM, Laurila A, Palo J, Merilä J 2004 Population differentiation in G matrix structure due to natural selection in *Rana temporaria*. Evolution 58, 2013–2020. (10.1111/j.0014-3820.2004.tb00486.x) 15521458

[13] Doroszuk A, Wojewodzic MW, Gort G, Kammenga JE 2008 Rapid divergence of genetic variance–covariance matrix within a natural population. Am. Nat. 171, 291–304. (10.1086/527478) 18271724

[14] Johansson F, Lind MI, Ingvarsson PK, Bokma F 2012 Evolution of the G-matrix in life history traits in the common frog during a recent colonisation of an island system. Evol. Ecol. 26, 863–878. (10.1007/s10682-011-9542-2)

[15] Relyea RA 2004 Fine-tuned phenotypes: tadpole plasticity under 16 combinations of predators and competitors. Ecology 85, 172–179. (10.1890/03-0169)

[16] Husby A, Nussey DH, Visser ME, Wilson AJ, Sheldon BC, Kruuk LEB 2010 Contrasting patterns of phenotypic plasticity in reproductive traits in two great tit (*Parus major*) populations. Evolution 64, 2221–2237. (10.1111/j.1558-5646.2010.00991.x) 20298465

[17] Dennis SR, Carter MJ, Hentley WT, Beckerman AP 2011 Phenotypic convergence along a gradient of predation risk. Proc. R. Soc. B 278, 1687–1696. (10.1098/rspb.2010.1989) PMC3081771 21084350

[18] Chun YJ, Collyer ML, Moloney KA, Nason JD 2007 Phenotypic plasticity of native vs. invasive purple loosestrife: a two-state multivariate approach. Ecology 88, 1499–1512. (10.1890/06-0856) 17601142

[19] Robinson MR, Beckerman AP 2013 Quantifying multivariate plasticity: genetic variation in resource acquisition drives plasticity in resource allocation to components of life history. Ecol. Lett. 16, 281–290. (10.1111/ele.12047) 23301600

[20] Scharloo W 1991 Canalization: genetic and developmental aspects. Annu. Rev. Ecol. Syst. 22, 65–93. (10.1146/annurev.es.22.110191.000433)

[21] Berger D, Grieshop K, Lind MI, Goenaga J, Maklakov AA, Arnqvist G 2014 Intralocus sexual conflict and environmental stress. Evolution 68, 2184–2196. (10.1111/evo.12528) 24766035

[22] Draghi JA, Whitlock MC 2012 Phenotypic plasticity facilitates mutational variance, genetic variance, and evolvability along the major axis of environmental variation. Evolution 66, 2891–2902. (10.1111/j.1558-5646.2012.01649.x) 22946810

[23] Schluter D 1996 Adaptive radiation along genetic lines of least resistance. Evolution 50, 1766–1774. (10.2307/2410734) 28565589

[24] Steppan SJ, Phillips PC, Houle D 2002 Comparative quantitative genetics: evolution of the G matrix. Trends Ecol. Evol. 17, 320–327. (10.1016/S0169-5347(02)02505-3)

[25] Arnold SJ, Bürger R, Hohenlohe PA, Ajie BC, Jones AG 2008 Understanding the evolution and stability of the G-matrix. Evolution 62, 2451–2461. (10.1111/j.1558-5646.2008.00472.x) 18973631 PMC3229175

[26] Eroukhmanoff F, Svensson EI 2011 Evolution and stability of the G-matrix during the colonization of a novel environment. J. Evol. Biol. 24, 1363–1373. (10.1111/j.1420-9101.2011.02270.x) 21507116

[27] Parejko K, Dodson SI 1991 The evolutionary ecology of an antipredator reaction norm: *Daphnia pulex* and *Chaoborus americanus*. Evolution 45, 1665–1674. (10.2307/2409787) 28564128

[28] Lynch M 1980 The evolution of cladoceran life histories. Q. Rev. Biol. 55, 23–42. (10.1086/411614)

[29] Riessen HP 1999 Predator-induced life history shifts in *Daphnia*: a synthesis of studies using meta-analysis. Can. J. Fish. Aquat. Sci. 56, 2487–2494. (10.1139/f99-155)

[30] Boeing WJ, Ramcharan CW, Riessen HP 2006 Multiple predator defence strategies in *Daphnia pulex* and their relation to native habitat. J. Plankton Res. 28, 571–584. (10.1093/plankt/fbi142)

[31] Tollrian R 1995 Predator-induced morphological defenses: costs, life history shifts, and maternal effects in *Daphnia pulex*. Ecology 76, 1691–1705. (10.2307/1940703)

[32] Beckerman AP, Rodgers GM, Dennis SR 2010 The reaction norm of size and age at maturity under multiple predator risk. J. Anim. Ecol. 79, 1069–1076. (10.1111/j.1365-2656.2010.01703.x) 20522144

[33] Taylor BE, Gabriel W 1992 To grow or not to grow: optimal resource allocation for *Daphnia*. Am. Nat. 139, 248–266. (10.1086/285326)

[34] Carpenter SR et al. 1987 Regulation of lake primary productivity by food web structure. Ecology 68, 1863–1876. (10.2307/1939878) 29357166

[35] Hammill E 2007 The ecology and evolution of refuges in an aquatic ecosystem: from inducible defences to habitat complexity. PhD thesis, University of Sheffield, Sheffield, UK.

[36] Brooks JL, Dodson SI 1965 Predation, body size, and composition of plankton. Science 150, 28–35. (10.1126/science.150.3692.28) 17829740

[37] Spitze K 1991 *Chaoborus* predation and life-history evolution in *Daphnia pulex*: temporal pattern of population diversity, fitness, and mean life history. Evolution 45, 82–92. (10.2307/2409484) 28564082

[38] ASTM. 1989 Standard guide for conducting acute toxicity tests with fishes, macroinvertebrates and amphibians. In Annual book of ASTM standards, pp. 379–397. West Conshohocken, PA: ASTM International.

[39] Beckerman A, Wieski K, Baird D 2007 Behavioural versus physiological mediation of life history under predation risk. Oecologia 152, 335–343. (10.1007/s00442-006-0642-6) 17219130

[40] Riessen HP, Sprules WG 1990 Demographic costs of antipredator defenses in *Daphnia pulex*. Ecology 71, 1536–1546 (10.2307/1938290)

[41] Hammill E, Rogers A, Beckerman AP 2008 Costs, benefits and the evolution of inducible defences: a case study with *Daphnia pulex*. J. Evol. Biol. 21, 705–715. (10.1111/j.1420-9101.2008.01520.x) 18355186

[42] Stearns SC 1992 The evolution of life histories. New York, NY: Oxford University Press.

[43] Strang G 2009 Introduction to linear algebra. Wellesley, MA: Wellesley-Cambridge Press.

[44] R Core Team. 2013 R: a language and environment for statistical computing. Vienna, Austria: R Foundation for Statistical Computing.

[45] Hadfield J 2010 MCMC methods for multi-response generalized linear mixed models: the MCMCglmm R package. J. Stat. Softw. 33, 1–22.20808728

[46] Lenth RV 2009 Response-surface methods in R, using rsm. J. Stat. Softw. 32, 1–17.

[47] Brooks R, Hunt J, Blows MW, Smith MJ, Bussiére LF, Jennions MD 2005 Experimental evidence for multivariate stabilizing sexual selection. Evolution 59, 871–880. (10.1111/j.0014-3820.2005.tb01760.x) 15926696

[48] Swift MC, Fedorenko AY 1975 Some aspects of prey capture by *Chaoborus* larvae. Limnol. Oceanogr. 20, 418–425. (10.2307/2835201)

[49] Pastorok R 1981 Prey vulnerability and size selection by *Chaoborus* larvae. Ecology 62, 1311–1324. (10.2307/1937295)

[50] O'Brien WJ 1979 The predator-prey interaction of planktivorous fish and zooplankton. Am. Sci. 67, 572–581. (10.2307/27849438)

[51] Lande R, Arnold SJ 1983 The measurement of selection on correlated characters. Evolution 37, 1210–1226. (10.2307/2408842) 28556011

[52] Ovaskainen O, Cano JM, Merilä J 2008 A Bayesian framework for comparative quantitative genetics. Proc. R. Soc. B 275, 669–678. (10.1098/rspb.2007.0949) PMC2596838 18211881

[53] Berger D, Postma E, Blanckenhorn WU, Walters RJ 2013 Quantitative genetic divergence and standing genetic (co)variance in thermal reaction norms along latitude. Evolution 67, 2385–2399. (10.1111/evo.12138) 23888859

[54] Stearns S, de Jong G, Newman B 1991 The effects of phenotypic plasticity on genetic correlations. Trends Ecol. Evol. 6, 122–126. (10.1016/0169-5347(91)90090-K) 21232440

[55] De Meester L 1993 Genotype, fish-mediated chemical, and phototactic behavior in *Daphnia magna*. Ecology 74, 1467–1474. (10.2307/1940075)

[56] De Meester L 1996 Evolutionary potential and local genetic differentiation in a phenotypically plastic trait of a cyclical parthenogen, *Daphnia magna*. Evolution 50, 1293–1298. (10.2307/2410669) 28565281

[57] Walsh MR, Post DM 2011 Interpopulation variation in a fish predator drives evolutionary divergence in prey in lakes. Proc. R. Soc. B 278, 2628–2637. (10.1098/rspb.2010.2634) PMC3136835 21270045

