# Supplementary material for: The alignment between phenotypic plasticity, the major axis of genetic variation and the response to selection
Source: Proc Biol Sci. 2015 Oct 7;282(1816):20151651. doi: 10.1098/rspb.2015.1651 (PMC4614775; doi:10.1098/rspb.2015.1651)
Supplement: Supplementary material [file rspb20151651supp1.pdf]

Supplementary materials

Lind MI, Yarlett K, Reger J, Carter MC and AP Beckerman (2015) The alignment between phenotypic plasticity, the major axis of genetic variation and the response to selection *Proc. R. Soc. B: Biol. Sci.*

Below is presented the microsatellite markers and methodology, followed by supplementary tables 1-10 and supplementary figures 1-4

Microsatellite markers and methodology

All clones in this study were screened at 10 polymorphic microsatellite markers developed by Cristescu et al. (2006) and Stapley et al. (unpublished). All DNA extractions were performed from whole adults by placing individual females in a 1.5 ml flip-top tube with 50 µl buffer (made up of 10 mM Tris-Cl pH 8.2, 1 mM EDTA and 25 mM NaCl) and 4 µl proteinase K, followed by an incubation period of one hour at 55°C and finally three minutes at 80°C to denature the proteinase K. Allelic variation was assessed using the following neutral markers arranged in three, 2 µl Qiagen Multiplex reactions: (i) Dp802; Dp1236, Dp1290; (ii) Dpu122, Dp1079, Dp675; and (iii) Dp1123, Dp45, Dp460, Dp43. We used a touchdown PCR to lower nonspecific amplification (for details see Cristescu et al. 2006). Amplified products were genotyped in an ABI 3730 48-well capillary DNA Analyser (Applied Biosystems) and allele sizes were scored using GENEMAPPER v3.7 software (Applied Biosystems).

Note: The microsatellite analyses were performed by Julia Reger and are presented in her PhD. Thesis (Reger 2012).

References

Cristescu, M. E. A., J. K. Colbourne, J. Radivojac, and M. Lynch. 2006. A microsatellite-based genetic linkage map of the waterflea, Daphnia pulex: On the prospect of crustacean genomics. Genomics 88:415–430.

Reger, J. 2012. The quantitative genetic basis of inducible defences and life-history plasticity in Daphnia pulex. Ph. D. Thesis. University of Sheffield, Department of Animal and Plant Sciences, Sheffield, United Kingdom.

Supplementary table 1. Pond names, locations, some physical and biological characteristics and the number of genotypes identified per pond.

Pond	Location	Coordinates	pH	Vegetation	Cover	No. genotypes
LD3	Cumbria	54°20′39.8791″N, 002°50′53.9422″W	8.50	Heavy	Light	5
LD6	Cumbria	54°20′51.8643″N, 002°53′07.1089″W	8.46	Present	Light	7
Crabtree	Yorkshire	53°24′18.4949″N, 001°27′27.7570″W	8.62	Heavy	Light	7

Supplementary table 2. Clone mean ± SE of traits in both predator cue treatments.

Trait	Treatment	Trait mean ± SE
Age at maturity	Fish	6.88 ± 0.18 days
	Midge	6.90 ± 0.13 days
Size at maturity	Fish	1.68 ± 0.02 mm
	Midge	1.86 ± 0.02 mm
Adult growth rate	Fish	0.0378 ± 0.0032 day^{-1}
	Midge	0.0327 ± 0.0025 day^{-1}

Supplementary table 3. Heritability (h^2), the genetic variance-covariance matrix (**G**) and genetic correlations between age at maturity, size at maturity and adult growth rate traits in the fish and midge cue treatment. Presented are the posterior mode and 95% highest posterior density (HPD) intervals estimated by MCMC. The **G** matrix is presented in bold, with genetic variance as diagonal elements, and genetic covariance as off-diagonal elements. Genetic correlations are presented as off-diagonal elements above the **G** matrix with non-zero estimates indicated by asterisks (*).

**Fish**	h^2	Age maturity	Size maturity	Adult growth
Age maturity	0.533* (0.305; 0.761)	**0.530* (0.239; 1.549)**	0.603* (0.029; 0.827)	-0.586 (-823; 0.038)
Size maturity	0.598* (0.373; 0.784)	**0.152 (-0.053; 0.561)**	**0.352* (0.133; 0.695)**	-0.850* (-0.964; -0.485)
Adult growth	0.695* (0.461; 0.825)	**-0.284 (-0.820; 0.121)**	**-0.330* (-0.837; -0.129)**	**0.657* (0.327; 1.517)**

**Midge**	h^2	Age maturity	Size maturity	Adult growth
Age maturity	0.332* (0.039; 0.600)	**0.171* (<0.001; 0.738)**	-0.371 (-0.843; 0.281)	0.360 (-0.271; 0.773)
Size maturity	0.302* (0.039; 0.551)	**-0.039 (-0.255; 0.096)**	**0.106* (<0.001; 0.492)**	-0.600* (-0.900; -0.060)
Adult growth	0.460* (0.267; 0.724)	**0.099 (-0.126; 0.354)**	**-0.117 (-0.464; 0.024)**	**0.316* (0.136; 0.869)**

Supplementary table 4. Eigendecomposition of **G** into constituent eigenvalues with cumulative variance explained and their corresponding eigenvectors and trait loadings in the fish and midge cue treatment.

	Fish			Midge		
Eigenvalue	1.127	0.450	0.120	0.415	0.125	0.053
Cumm. expl.	66.40%	26.54%	7.06%	69.95%	21.08%	8.97%
	1	2	3	1	2	3
Age maturity	- 0.503	0.830	- 0.239	0.400	0.915	-0.052
Size maturity	- 0.475	- 0.035	0.880	-0.369	0.212	0.905
Adult growth	0.722	0.556	0.412	0.839	-0.342	0.423

Supplementary table 5. The linear selection gradient β, the matrix of non-linear selection gradients, γ, and eigenvalues (m_i) and their corresponding eigenvectors from a canonical rotation of γ. Significant components of β, γ and significant eigenvalues after permutation are indicated by an asterisk (*).

Fish

Trait	β	γ -matrix Age maturity	Size maturity	Adult growth	m_i	γ_i	Age maturity	Size maturity	Adult growth
Age maturity	-0.762*	0.256			m_1	0.195	0.915	-0.228	-0.334
Size maturity	0.547*	-0.222	-0.271		m_2	-0.008	0.137	-0.602	0.787
Adult growth	0.595*	-0.218	-0.157	0.099	m_3	-0.244	-0.380	-0.765	-0.519

Midge

Trait	β	γ -matrix Age maturity	Size maturity	Adult growth	m_i	γ_i	Age maturity	Size maturity	Adult growth
Age maturity	-0.743*	0.341			m_1	0.257	0.854	-0.501	0.139
Size maturity	0.515*	-0.247	0.151		m_2	0.188*	0.188	0.547	0.816
Adult growth	0.472*	0.174	0.208	0.196	m_3	-0.101	-0.485	-0.671	0.562

Supplementary table 6. One-tailed p values after permutation for the linear selection gradient β, the matrix of non-linear selection gradients, γ and eigenvalues (m_i) from a canonical rotation of γ.

Fish

		γ -matrix					
Trait	β	Age maturity	Size maturity	Adult growth	DVM	m_i	γ_i
Age maturity	>0.999*	0.050				m_1	0.674
Size maturity	<0.001*	0.872	0.800			m_2	0.560
Adult growth	<0.001*	0.911	0.813	0.664		m_3	0.455

Midge

		γ -matrix					
Trait	β	Age maturity	Size maturity	Adult growth	DVM	m_i	γ_i
Age maturity	>0.999*	0.036				m_1	0.496
Size maturity	<0.001*	0.973	0.296			m_2	0.020
Adult growth	<0.001*	0.108	0.186	0.262		m_3	0.136

Supplementary table 7. The trait-specific coefficients for the composite selection gradients. Each gradient is standardized to a length of 1.

Treatment	Trait	β_R	β_R+0.5β_S	β_R+ β_S	0.5β_R+ β_S	β_S
Fish	Age at maturity	-0.686	-0.788	-0.681	-0.394	0.000
	Size at maturity	0.492	-0.009	-0.504	-0.866	-1.000
	Adult growth	0.536	0.616	0.532	0.308	0.000
Midge	Age at maturity	-0.728	-0.550	-0.417	-0.275	0.000
	Size at maturity	0.505	0.759	0.867	0.945	1.000
	Adult growth	0.463	0.350	0.267	0.175	0.000

Supplementary table 8. Tests for differences in angle between the predicted response to selection (Δz) in the fish and midge cue treatment. The comparisons relates to selection gradients (β) with different weighting of reproductive and survival selection. Angle is expressed as the posterior mode with 95% confidence interval.

Selection response test	Angle	p
fish vs. midge: $\Delta z_{\beta(R)}$	48.92° (11.59°-89.35°)	0.062
fish vs. midge: $\Delta z_{\beta(R)+0.5\beta(S)}$	77.69° (35.43°-118.70°)	<0.001
fish vs. midge: $\Delta z_{\beta(R)+\beta(S)}$	99.39° (51.61°-133.39°)	<0.001
fish vs. midge: $\Delta z_{0.5\beta(R)+\beta(S)}$	107.06° (66.47°-149.00°)	<0.001
fish vs. midge: $\Delta z_{\beta(S)}$	137.83° (100.38°-172.13°)	<0.001

Supplementary table 9. The response to selection (Δz) using composite selection gradients based upon different weighting of reproduction (β_R) and survival (β_S) selection. The multivariate response to selection is partitioned into trait-specific direct (through genetic variance), indirect (through genetic covariance) and total (using all components of **G**) response in the fish and midge cue treatment. Posterior mode and the upper and lower bound of the 95% credibility interval are presented, and elements significantly different from zero are indicated by asterisks (*).

Fish

Response	Trait	Total response	Direct selection	Indirect selection
$\Delta z_{\beta(R)}$	Age maturity	-0.421* (-1.188; -0.197)	-0.437* (-1.050; -0.156)	-0.056 (-0.228; 0.091)
$\Delta z_{\beta(R)+0.5\beta(S)}$	Age maturity	-0.591* (-1.511; -0.230)	-0.417* (-1.165; -0.188)	-0.141 (-0.500; 0.056)
$\Delta z_{\beta(R)+\beta(S)}$	Age maturity	-0.610* (-1.643; -0.267)	-0.405* (-1.032; -0.155)	-0.217 (-0.717; 0.046)
$\Delta z_{0.5\beta(R)+\beta(S)}$	Age maturity	-0.362* (-1.217; -0.162)	-0.208* (-0.582; -0.094)	-0.176 (-0.734; 0.035)
$\Delta z_{\beta(S)}$	Age maturity	-0.196 (-0.593; 0.028)	0.000 (0.000; 0.000)	-0.196 (-0.593; 0.028)
$\Delta z_{\beta(R)}$	Size maturity	-0.138 (-0.498; 0.038)	0.174* (0.068; 0.345)	-0.265* (-0.767; -0.100)
$\Delta z_{\beta(R)+0.5\beta(S)}$	Size maturity	-0.309* (-0.879; -0.129)	-0.003* (-0.006; -0.001)	-0.303* (-0.873; -0.126)
$\Delta z_{\beta(R)+\beta(S)}$	Size maturity	-0.409* (-1.087; -0.224)	-0.175* (-0.352; -0.073)	-0.259* (-0.754; -0.114)
$\Delta z_{0.5\beta(R)+\beta(S)}$	Size maturity	-0.437* (-0.998; -0.199)	-0.270* (-0.602; -0.118)	-0.152* (-0.436; -0.063)
$\Delta z_{\beta(S)}$	Size maturity	-0.353* (-0.700; -0.138)	-0.353* (-0.700; -0.138)	0.000 (0.000; 0.000)
$\Delta z_{\beta(R)}$	Adult growth	0.415* (0.122; 0.932)	0.350* (0.175; 0.813)	0.004 (-0.275; 0.295)
$\Delta z_{\beta(R)+0.5\beta(S)}$	Adult growth	0.732* (0.252; 1.461)	0.404* (0.191; 0.915)	0.230 (-0.068; 0.638)
$\Delta z_{\beta(R)+\beta(S)}$	Adult growth	0.895* (0.313; 1.625)	0.351* (0.174; 0.789)	0.382* (0.082; 0.923)
$\Delta z_{0.5\beta(R)+\beta(S)}$	Adult growth	0.580* (0.246; 1.377)	0.202* (0.096; 0.458)	0.411* (0.132; 0.961)
$\Delta z_{\beta(S)}$	Adult growth	0.329* (0.131; 0.843)	0.000 (0.000; 0.000)	0.329* (0.131; 0.843)

Midge

Response	Trait	Total response	Direct selection	Indirect selection
$\Delta z_{\beta(R)}$	Age maturity	-0.123 (-0.538; 0.003)	-0.116* (-0.538; -0.000)	0.008 (-0.081; 0.090)
$\Delta z_{\beta(R)+0.5\beta(S)}$	Age maturity	-0.168 (-0.430; 0.007)	-0.117* (-0.406; -0.000)	-0.030 (-0.115; 0.074)
$\Delta z_{\beta(R)+\beta(S)}$	Age maturity	-0.118* (-0.373; -0.001)	-0.071* (-0.308; -0.000)	-0.038 (-0.163; 0.078)
$\Delta z_{0.5\beta(R)+\beta(S)}$	Age maturity	-0.117 (-0.326; 0.007)	-0.058* (-0.213; -0.000)	-0.46 (-0.191; 0.076)
$\Delta z_{\beta(S)}$	Age maturity	-0.039 (-0.249; 0.103)	0.000 (0.000; 0.000)	-0.039 (-0.249; 0.103)
$\Delta z_{\beta(R)}$	Size maturity	0.065 (-0.044; 0.256)	0.059* (0.000; 0.250)	0.004 (-0.185; 0.110)
$\Delta z_{\beta(R)+0.5\beta(S)}$	Size maturity	0.129* (0.005; 0.345)	0.091* (0.005; 0.373)	-0.003 (-0.141; 0.086)
$\Delta z_{\beta(R)+\beta(S)}$	Size maturity	0.104 (-0.006; 0.391)	0.102* (0.000; 0.430)	0.000 (-0.107; 0.064)
$\Delta z_{0.5\beta(R)+\beta(S)}$	Size maturity	0.106* (0.022; 0.452)	0.114* (0.007; 0.465)	-0.002 (-0.070; 0.043)
$\Delta z_{\beta(S)}$	Size maturity	0.117* (0.000; 0.494)	0.117* (0.000; 0.494)	0.000 (0.000; 0.000)
$\Delta z_{\beta(R)}$	Adult growth	0.037 (-0.137; 0.286)	0.177* (0.063; 0.402)	-0.074 (-0.393; 0.039)
$\Delta z_{\beta(R)+0.5\beta(S)}$	Adult growth	-0.005 (-0.252; 0.149)	0.110* (0.048; 0.299)	-0.125 (-0.456; 0.027)
$\Delta z_{\beta(R)+\beta(S)}$	Adult growth	-0.041 (-0.309; 0.104)	0.084* (0.036; 0.236)	-0.135 (-0.484; 0.017)
$\Delta z_{0.5\beta(R)+\beta(S)}$	Adult growth	-0.074 (-0.366; 0.071)	0.055* (0.024; 0.150)	-0.117 (-0.501; 0.002)
$\Delta z_{\beta(S)}$	Adult growth	-0.139 (-0.469; 0.024)	0.000 (0.000; 0.000)	-0.139 (-0.469; 0.024)

Supplementary table 10. Validating the power to detect mis-alignment. Tests for alignment between the plasticity vector induced by the opposite predator cue treatment, the direction of maximum genetic variation (g_{max}) and the response to selection on reproduction (Δz_R), survival (Δz_S) or reproduction and survival combined. Angle is expressed as the posterior mode with 95% confidence interval. Significant angles indicate mis-alignment.

Comparison	Angle	p	Alignment
$Plasticity_{midge}$, $g_{max\ fish}$	92.90° (56.15°-135.10°)	0.169	yes
$Plasticity_{midge}$, $\Delta z_{\beta(R)\ fish}$	90.61° (61.37°-131.07°)	<0.001	-
$Plasticity_{midge}$, $\Delta z_{\beta(R)+0.5\beta(S)\ fish}$	90.16° (67.57°-133.39°)	<0.001	-
$Plasticity_{midge}$, $\Delta z_{\beta(R)+\beta(S)\ fish}$	100.54° (70.12°-136.65°)	<0.001	-
$Plasticity_{midge}$, $\Delta z_{0.5\beta(R)+\beta(S)\ fish}$	102.79° (73.52°-142.00°)	<0.001	-
$Plasticity_{midge}$, $\Delta z_{\beta(S)\ fish}$	116.96° (77.39°-148.76°)	<0.001	-
$Plasticity_{fish}$, $g_{max\ midge}$	61.02° (19.43°-118.04°)	0.084	yes
$Plasticity_{fish}$, $\Delta z_{\beta(S)\ midge}$	104.94° (56.59°-152.52°)	<0.001	-
$Plasticity_{fish}$, $\Delta z_{\beta(R)+0.5\beta(S)\ midge}$	122.51° (75.87°-166.28°)	<0.001	-
$Plasticity_{fish}$, $\Delta z_{\beta(R)+\beta(S)\ midge}$	131.96° (81.83°-170.56°)	0.001	-
$Plasticity_{fish}$, $\Delta z_{0.5\beta(R)+\beta(S)\ midge}$	141.62° (92.57°-171.72°)	<0.001	-
$Plasticity_{fish}$, $\Delta z_{\beta(S)\ midge}$	147.70° (103.59°-174.65°)	<0.001	-

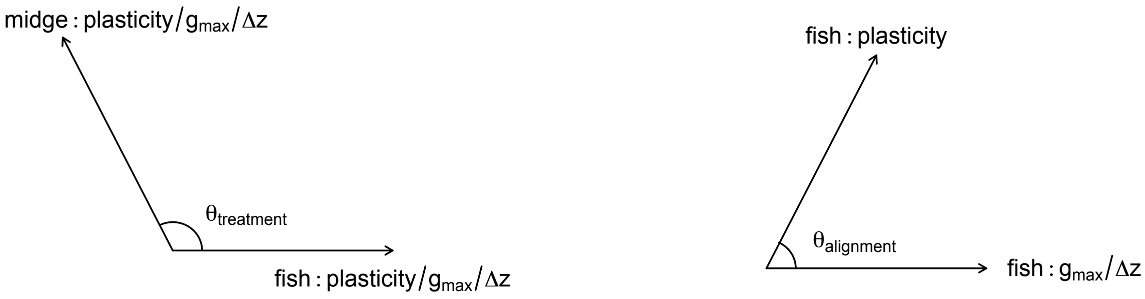

Supplementary figure 1. Specific hypothesis tested and their interpretation.

Angle comparison	Interpretation
$plasticity_{fish}$ vs. $plasticity_{midge}$	Difference in the direction of plastic trait induction between treatments
$g_{max(fish)}$ vs. $g_{max(midge)}$	Difference in the direction of maximum genetic variance between treatments
$\beta_{R/S(fish)}$ vs. $\beta_{R/S(midge)}$	Difference in selection gradient (reproduction /survival) between treatments
$\Delta z_{R/S(fish)}$ vs. $\Delta z_{R/S(midge)}$	Difference in the predicted response to selection between treatments
g_{max} vs. plasticity	Alignment between g_{max} and plasticity in each predator treatment
$\Delta z_{R/S}$ vs. plasticity	Alignment between the predicted response to selection and plasticity in each predator treatment

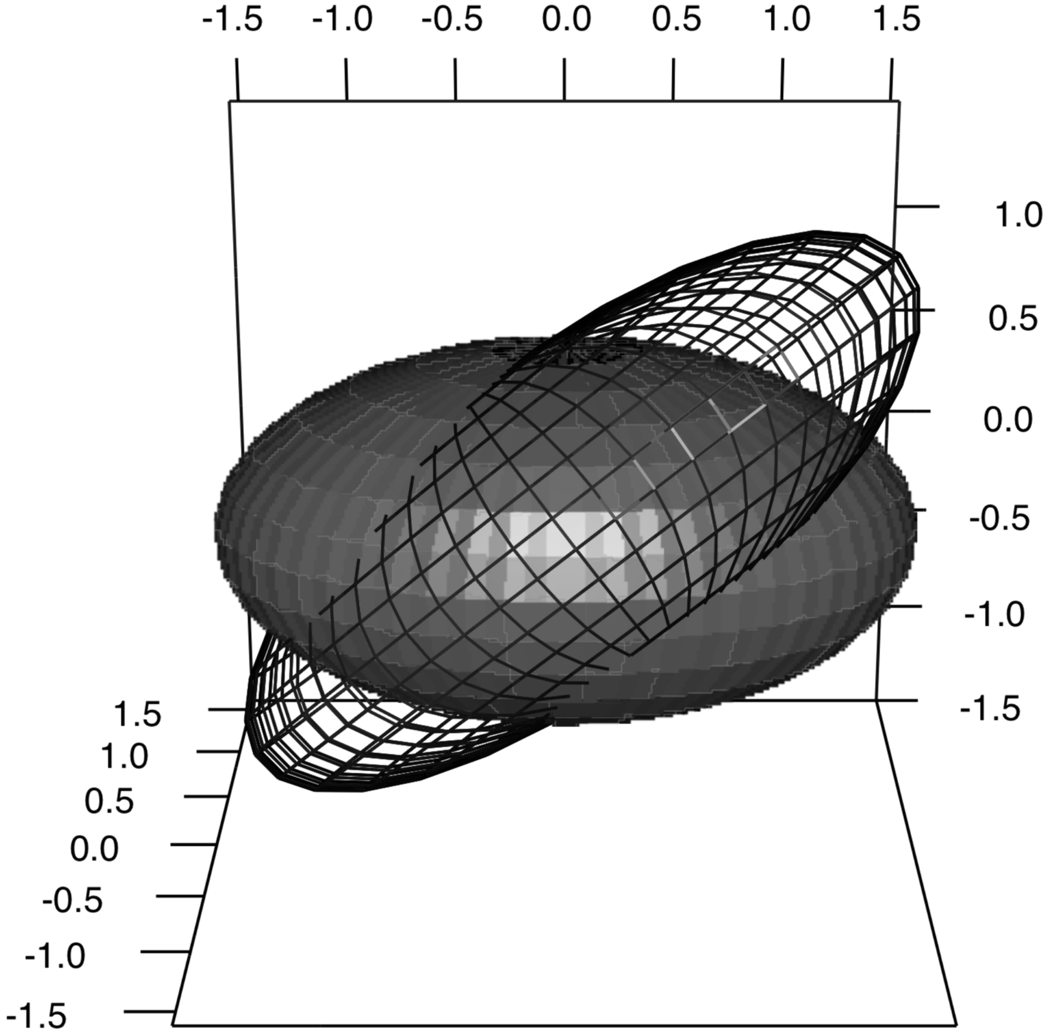

Supplementary figure 2. Three-dimensional ellipsoids representing the first three eigenvectors of the genetic variance-covariance matrices in the midge (grey, solid surface) and fish (black, wireframe) cue treatment.

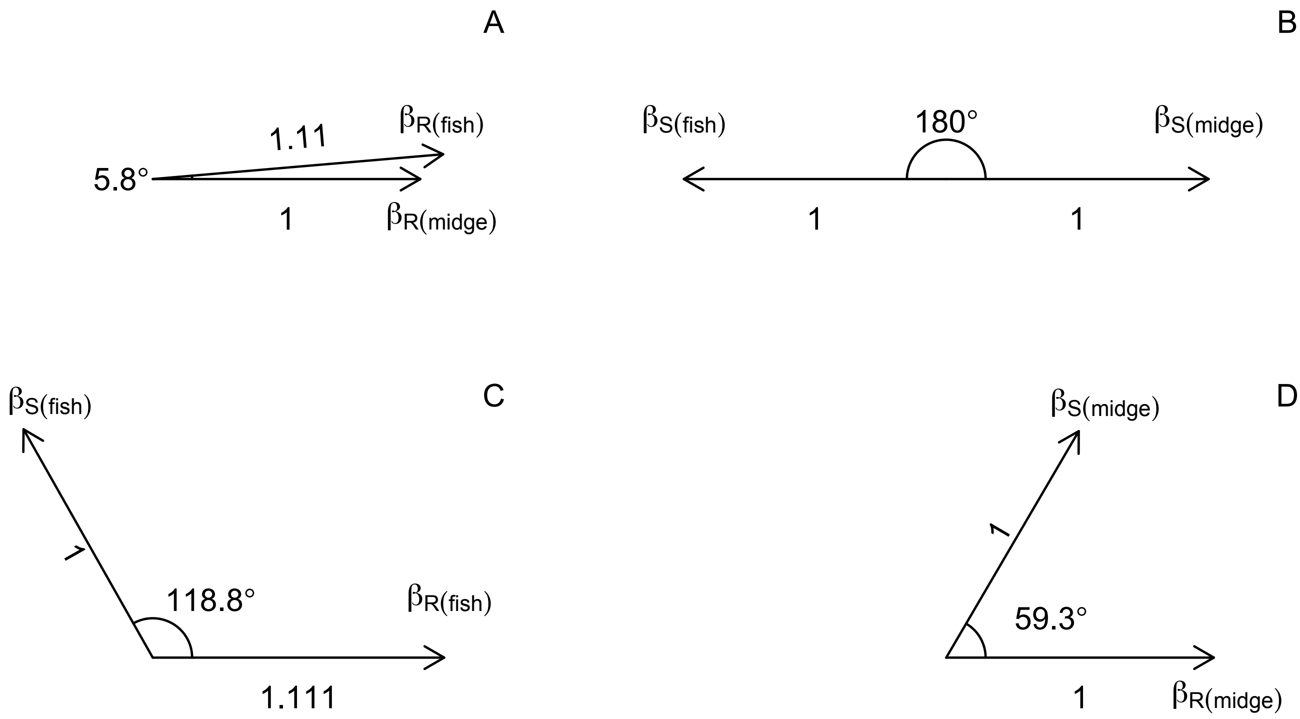

Supplementary figure 3. The difference in magnitude and orientation of the vector of the linear selection gradient (β) visualised in two-dimensional space when comparing (A) reproduction or (B) survival in the midge and fish treatment. The difference in angle between β in reproduction and survival is presented separately for (C) the fish and (D) the midge cue treatment. The length of each vector illustrates the total strength of selection.

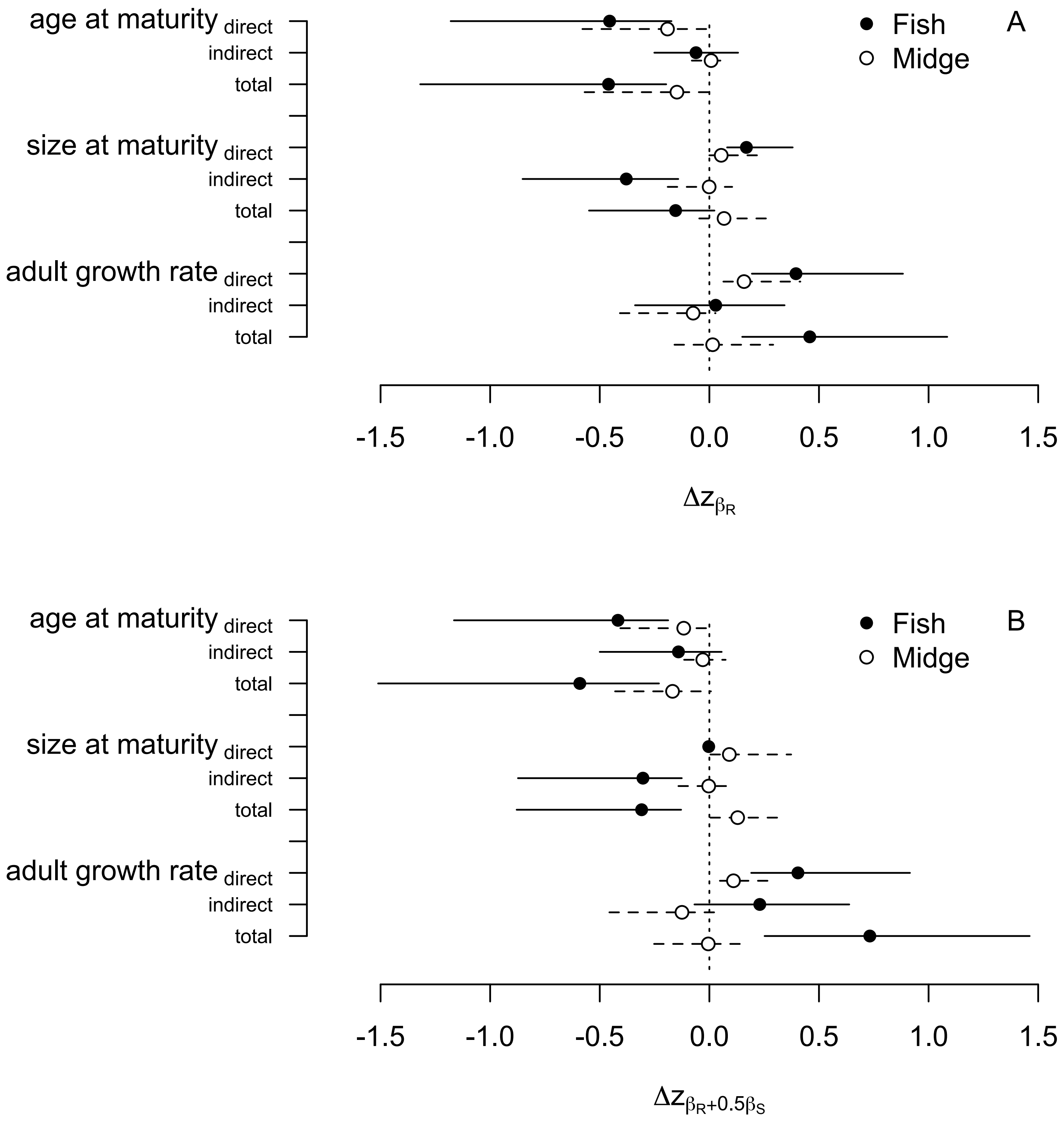
age at maturity direct
indirect
total
size at maturity direct
indirect
total
adult growth rate direct
indirect
total
Fish
Midge
A
-1.5
-1.0
-0.5
0.0
0.5
1.0
1.5
Δz_{β_R}
B
$\Delta z_{\beta_R + 0.5\beta_S}$

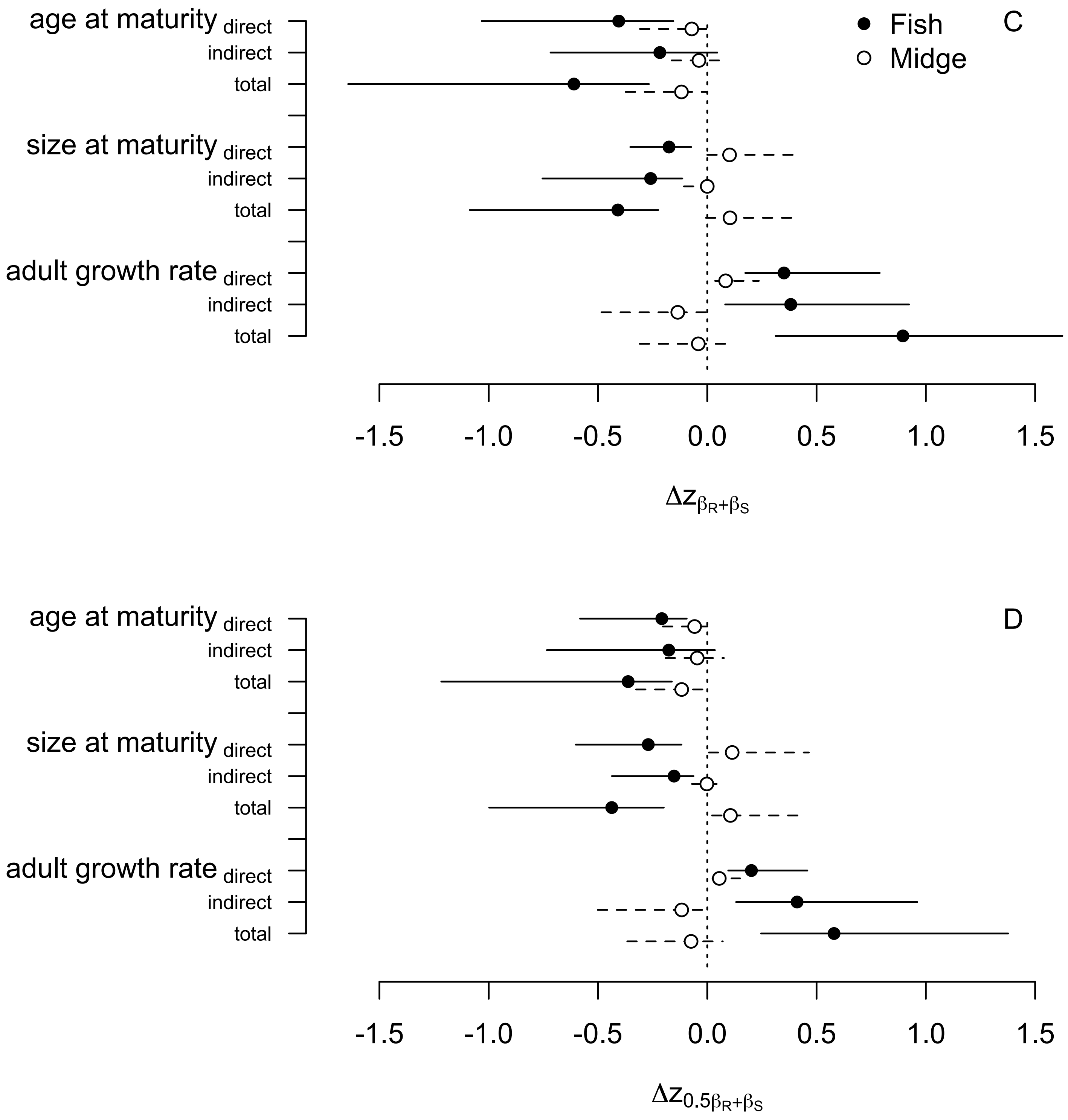
age at maturity direct
indirect
total
size at maturity direct
indirect
total
adult growth rate direct
indirect
total
Fish
Midge
C
-1.5
-1.0
-0.5
0.0
0.5
1.0
1.5
$\Delta z_{\beta_R+\beta_S}$
D
$\Delta z_{0.5\beta_R+\beta_S}$

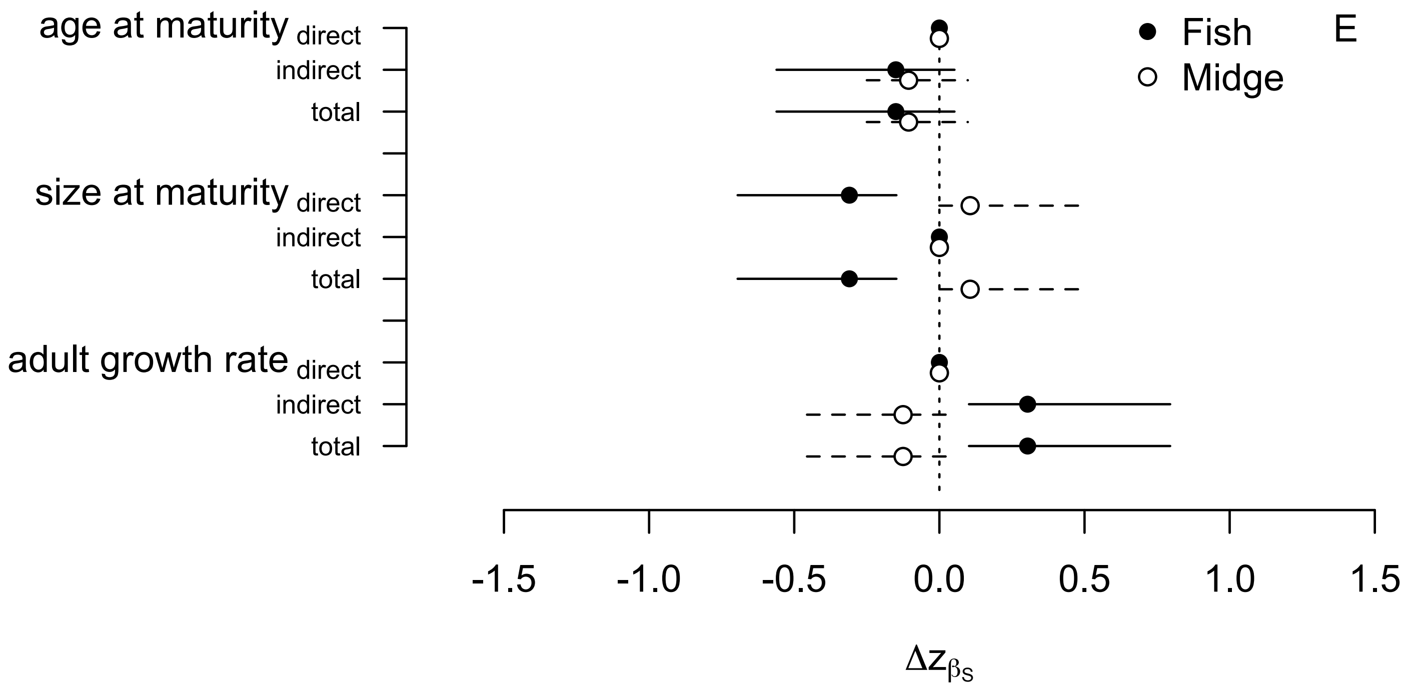

Supplementary figure 4. The predicted response to selection (Δz) using selection based on different combinations of reproductive and survival selection. The multivariate response to selection is partitioned into trait-specific direct (through genetic variance), indirect (through genetic covariance) and total (using all components of G) response to selection in the fish (black circles, solid lines) and midge (white circles, jagged lines) cue treatment. The horizontal lines correspond to the 95% HPD interval.
